# Enhanced expression and phosphorylation of Sirt7 activates smad2 and ERK signaling and promotes the cardiac fibrosis differentiation upon angiotensin-II stimulation

**DOI:** 10.1371/journal.pone.0178530

**Published:** 2017-06-05

**Authors:** Haichen Wang, Shengwu Liu, Shengqiang Liu, Wei Wei, Xiaolong Zhou, Fang Lin, Juanjuan Wang, Jinye Chen, Guodong Zhang, Yongbing Pang

**Affiliations:** 1 Department of Cardiovascular Surgery, First Affiliated Hospital of Xi'an Jiaotong University, Xi’an, Shaanxi, China; 2 Department of Neurology, Affiliated Hospital of Yan'an University, Yan'an, Shaanxi, China; 3 Department of Cardiology, Ankang Central Hospital, Ankang, Shaanxi, China; 4 Department of Neurosurgery, Affiliated Hospital of Yan'an University, Yan'an, Shaanxi, China; 5 Department of Neurosurgery, Baoji Central Hospital, Baoji, Shaanxi, China; 6 Department of Geriatrics, Affiliated Hospital of Yan'an University, Yan'an, Shaanxi, China; 7 Department of Cardiovascular and Pulmonary, Ankang Central Hospital, Ankang, Shaanxi, China; 8 Department of Brain Tumor, Baoji Central Hospital, Baoji, Shaanxi, China; Max Delbruck Centrum fur Molekulare Medizin Berlin Buch, GERMANY

## Abstract

Cardiac fibroblasts (CFs) phenotypic conversion to myofibroblasts (MFs) represents a crucial event in cardiac fibrosis that leads to impaired cardiac function. However, regulation of this phenotypic transformation remains unclear. Here, we showed that sirtuin-7 (Sirt7) plays an important role in the regulation of MFs differentiation. Sirt7 expression and phosphorylation were upregulated in CFs upon angiotensin-II (Ang-II) stimulation. Sirt7 depletion by siRNA in CFs resulted in decreased cell proliferation and extracellular matrix (ECM) deposition. Further, examination of Sirt7-depleted CFs demonstrated significantly lower expression of α-smooth muscle actin (α-SMA), the classical marker of MFs differentiation, and decreased formation of focal adhesions. Moreover, overexpression of Sirt7 increased α-SMA expression in Ang-II treated CFs and exacerbated Ang-II-induced MFs differentiation. Moreover, Sirt7 depletion could largely reverse Ang-II induced increase of nuclear translocalization and activity of smad2 and extracellular regulated kinases (ERK) in CFs. Importantly, the increased differentiation of CFs to MFs was also abolished by smad2 siRNA or U0126. Our findings reveal a novel role of Sirt7 and its phosphorylation in the phenotypic conversion of CFs to MFs and might lead to the development of new therapeutic and prognostic tools for cardiac fibrosis.

## 1. Introduction

Heart disease affects increasingly people all over the world [1]. In some forms of heart disease, including myocardial infarction, cardiomyopathies and hypertensive heart disease, fibrosis constitutes one of the most important responses of the heart to some pathological stimuli [2]. In the occurrence and development of cardiac fibrosis, cardiac fibroblasts (CFs) and their internal regulating signaling pathways play an important role.

CFs consist of approximately 60% of the cardiac cells population and play a key role as mediators in normal and pathological cardiac remodeling [3]. The persistent activation of CFs is identified by increased proliferation and subsequent extracellular matrix (ECM) secretion which contribute to maintain the structural integrity of heart [4]. Despite the fact that these changes are an important reparative wound healing response, they may become a prime cause for the accumulation of collagen and cardiac fibrosis if they last for a prolonged activation [5]. Therefore, many studies pointed specifically on its role in fibrosis [6]. In the process of promoting cardiac fibrosis, CFs could differentiate into myofibroblasts (MFs), a cell type which can secrete ECM components, that produces Ang-II and a variety of fibrogenic growth factors (FGF) and transforming-growth factor β (TGF-β) [7]. Furthermore, MFs can express contractile proteins, including α-smooth muscle actin (α-SMA), indicating acquisition of MFs phenotype [8].

Researches have demonstrated that CFs expressed some specific integrins, in which collagen and fibronectin are the primary ligand [9]. Not only integrin participate in the process, but also Ang-I receptor has been shown to be mechanically activated both in vitro and in vivo in the absence of angiotensin-II (Ang-II) [10] resulting in increasing ECM components, matrix metalloproteinases (MMPs) and integrin signaling in CFs [11]. Although there are many studies focused on cardiac fibrosis, the molecular mechanisms regulating cardiac fibrosis are to a large extent poorly understood.

Recently, the role of sirtuin in health and disease has been extensively studied. The sirtuin gene family was originally found in yeast as silent information regulator 2, Sir2 [12]. Sirtuins are a homologue of Sir2 in mammals, and its family consists of seven isoforms sirtuin1~7 (Sirt1 ~ 7). Sirtuins play an important role in many biological processes such as metabolism, cell survival, longevity and stress resistance. Among the seven isoforms, several members participate in the process of tissue fibrosis. Research has demonstrated that Sirt1 and Sirt2 suppressed renal fibrogenesis by suppressing phosphorylation of signal transducer and activator of transcription 3 (STAT3), epidermal growth factor receptor (EGFR) and platelet-derived growth factor receptor (PDGFR) [13]. Sirt1 was also shown to inhibit the tissue fibrosis by deacteylating smad4 and repressing the effect of TGF-β signaling on matrix metalloproteinase-7 (MMP7) [14]. In isolated CFs, the activation of Sirt3 by RSV suppressed CFs to MFs transformation via inhibition of the TGF-β/Smad3 pathway in response to Ang-II [15]. Moreover, Sirt6 inhibits CFs to MFs transformation via inactivation of nuclear factor κB (NF-κB) signaling [16].

Among the sirtuins isoforms, Sirt7 is the only sirtuin localized predominantly in the nucleoli and regulates RNA polymerase I transcription. In vitro, Sirt7-/- mouse or Sirt7 siRNA treated CFs showed reduced TGF-β signaling activation and low expression levels of fibrosis-related genes compared with wild-type mice or control siRNA treated cells [17]. Because TGF-β also promotes the phenotypic conversion of CFs to pathological MFs, which express α-SMA and produce ECM [18]. In addition to being associated with the TGF-β signaling pathway, Sirt7 could enhance mitogen-activated protein kinase (MAPK) pathway activity concomitantly with phosphorylated extracellular regulated kinases (p-ERK) in colorectal cancer [19]. While p-ERK not only can be a key molecule of some key signaling pathways, but also into the nucleus involved in the transcriptional regulation of many genes [20]. Moreover, there are studies shown that energy starvation induced an Sirt7 phosphorylation and subsequent subcellular redistribution, thereby further reducing rDNA transcription to overcome cell death [21]. This suggests that phosphorylation of Sirt7 plays an important role in its distribution and activation, but there is no more research on Sirt7 phosphorylation. Thus, these implied that Sirt7 may be involved in the pathogenesis of cardiac fibrosis, and participate in the regulation of TGF-β and ERK signal pathway. However, the precise role of Sirt7 in cardiac fibrosis remains unknown. Whether Sirt7 participates in the regulation of cardiac fibrosis through TGF-β signaling pathway and/or ERK pathway and the changes of its phosphorylation level play a role in cardiac fibrosis are both unclear. This study aims to investigate the possible roles of Sirt7 and phosphorylated Sirt7 in the regulation of MFs differentiation and to further explore the potential signaling pathway and molecular mechanisms in cardiac fibrosis.

## 2. Materials and methods

### 2.1. Cell and cell culture

The rat CFs were obtained from the Cell Biology Institute of Chinese Academy of Sciences (Shanghai, China). The CFs were cultured in Dulbecco’s modified Eagle’s medium containing 10% (v/v) new born calf serum (Gibco, Thermo Scientific, Grand Island, NY, USA) in a humidified incubator at 37°C with 5% CO2. Cells were grown on plastic dishes for protein extraction.

### 2.2. Plasmids and siRNAs

Full-length Sirt7 was amplified from cDNA. The polymerase chain reaction (PCR) products were cloned into the pEGFP-N3 (Beyotime, Nantong, China). The cells were seeded in 6-well plates, cultured to 80~90% confluence, and then transiently transfected with the plasmid by using Lipofectamine 3000 (Invitrogen) according to the reverse transfection method provided by the manufacturer.

Duplex oligonucleotides were chemically synthesized and purified by GenePharma (Shanghai, China). The small interfering RNA (siRNA) duplexes used were Sirt7, #1, 5’-CACCUUUCUGUGAGAACGGAA-3’, #2, 5’-UAGCCAUUUGUCCUUGAGGAA-3, and #3, 5’-CGCCAAAUACUUGGUCGUCUA-3. These three kinds of siRNAs were mixed (siRNA pool) to achieve the interference effect while eliminating the off target effect through reducing the use of each kind of siRNA. Cells were transfected with siRNA duplexes using Lipofectamine 3000 (Invitrogen) according to the reverse transfection method provided by the manufacturer.

### 2.3. Reagents and antibodies

Ang-II and U0126 were purchased from R&D systems (Minneapolis, MN, USA). Sirt7, GAPDH, α-SMA, paxillin, phospho-Smad2, phospho-Smad3, Smad2/3, GFP and Vinculin antibodies were purchased from Cell Signaling Technology (Danvers, MA, USA). LaminB, PAI-1, FN, P-Thr/Ser and Collagen I antibodies were purchased from Santa Cruz Biotechnology (Santa Cruz, CA, USA). HRP-conjugated secondary antibodies were purchased from Santa Cruz Biotechnology.

### 2.4. Western blotting analysis

Cytoplasmic and nuclear proteins were obtained using the Cytoplasmic and Nuclear Protein Extraction Kit (Sigma, USA) according to the manufacturer's instructions. Briefly, equal amounts of protein were run on SDS polyacrylamide gels and transferred to nitrocellulose membrane. The resulting blots were blocked with 5% non-fat dry milk and probed with antibodies. Then protein bands were detected by incubating with HRP-conjugated secondary antibodies (Santa Cruz, CA, USA) for 1–2 h at room temperature and visualized with ECL reagent (Thermo Scientific, Grand Island, NY, USA) by ImageQuant LA4000 mini imaging system (GE Healthcare). Densitometry analysis was performed using ImageJ software, and band intensities were normalized to those of GAPDH.

### 2.5. Immunoprecipitation

Cells were lysed with cell lysis buffer (20 mM Tris PH7.5, 150 mM NaCl, 1% Triton X-100, 2.5 mM sodium pyrophosphate, 1 mM EDTA, 1% Na3VO4, 0.5 μg/ml leupeptin, 1 mM PMSF). For coprecipitation assays, 500 μg of cell lysates were subjected to immunoprecipitation, and proteins precipitated were detected by western blot, as described previously (22). Antibodies against Sirt7 was used at dilutions of 1:100 for immunoprecipitation.

### 2.6. Immunofluorescence microscopy

Immunofluorescent was performed as previously described [22]. Briefly, cells were treated with or without 100 nM Ang-II for the time indicated after serum starvation overnight, fixed in ice-cold 4% paraformaldehyde (PFA) for 20min, rinsed with phosphate-buffered saline (PBS) for three times and permeabilized with 0.1% Triton X-100 before blocking in 1% BSA for 1 h. The cells were incubated with primary antibodies at 4°C overnight, and then incubated with secondary antibodies for 1.5h at room temperature. After wash with PBS, the samples were mounted with DAPI Fluoromount G (Southern Biotech, Birmingham, AL). Images were representatives of three independent experiments.

### 2.7. Cell viability assay

Cells were seeded in a 96-well plate as a density of 5000 cells/well for 24 h. Cell viability was measured using the Cell Counting Kit-8 (Dojindo Molecular Technologies, Japan), according to the manufacturer’s instructions. Absorbance was determined using the Multi-Mode Microplate Reader (PerkinElmer, Finland).

### 2.8. Hydroxyproline assay

The content of hydroxyproline in CFs culture supernatant was measured by a commercial kit (Nanjing Jiancheng Bioengineering Institute, Nanjing, China) according to the manufacturer’s instructions.

### 2.9. Luciferase report assay

In brief, CFs were seeded in 24-well plates 24 h before transfection. And then, the SBE reporter plasmid along with the internal control thymidine kinase promoter-Renilla luciferase reporter plasmid were co-transfected using lipofectamine 3000. After 8 hours of incubation, the cells were treated with Ang-II and collected for the luciferase assay using the Dual-Luciferase Reporter Assay System (Promega, Madison, Wisconsin) according to the manufacturer’s protocol. The luciferase activity was measured using the Relative luciferase activity was calculated as the ratio of firefly luciferase activity to Renilla luciferase activity. Experiments were performed in triplicates and repeated at least three times.

### 2.10. Statistical analysis

Data were presented as mean±standard error of the mean (SD). Statistical analyses were performed using GraphPad Prism 5.0 software (GraphPad Software, USA). Student’s t-test was used for comparison between two groups, and analysis of variance was used for comparison among groups. Values of P<0.05 were considered statistically significant. All experiments were repeated at least three times.

## 3. Results

### 3.1. Increased expression and activity of Sirt7 regulates CFs to MFs differentiation upon Ang -II stimulation

**Fig 1:**
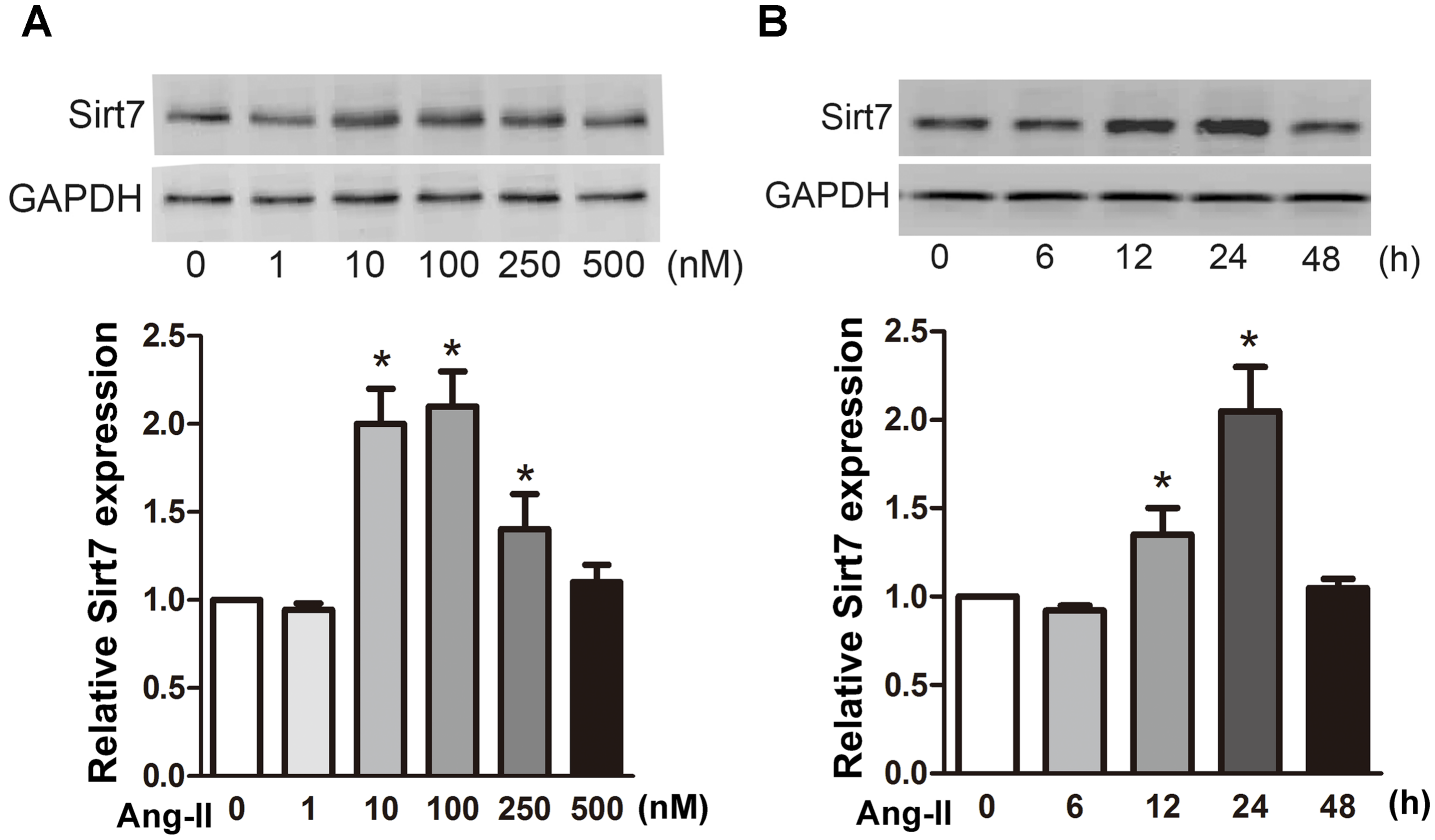
Ang-II regulates the expression of Sirt7 in CFs. (A) CFs were treated with Ang-II for the indicated concentration or (B) time. The expression of Sirt7 was analyzed by western blot. All experiments were repeated at least three times. (*P<0.05)

**Fig 2:**
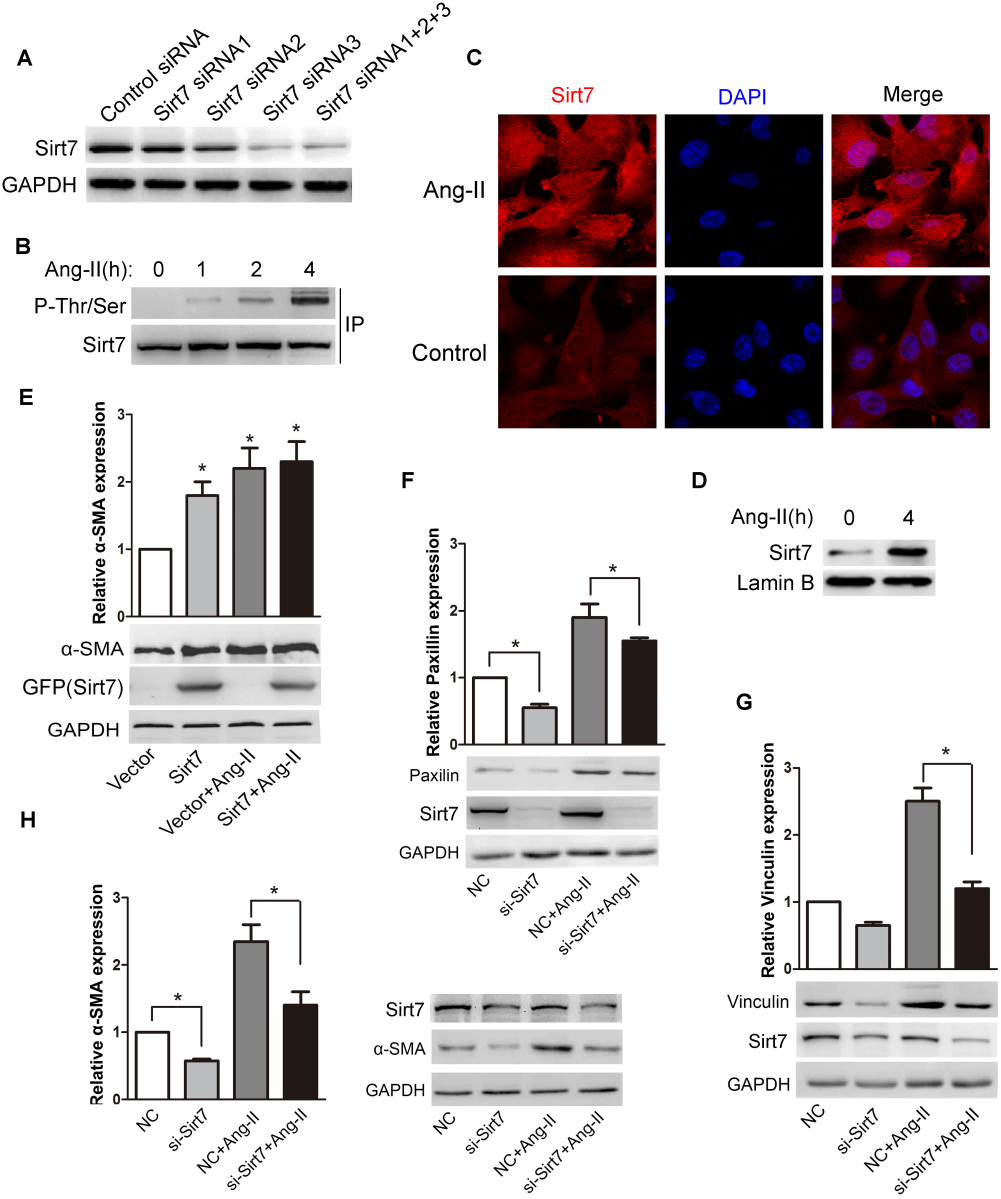
Increased expression and activity of Sirt7 regulates CFs fibrosis upon Ang-II stimulation. (A) CFs were transfected with Sirt7 siRNAs pool (si-Sirt7) and negative control (NC) siRNA. At 48 h after transfection, cells were lysed and subjected to western blot analysis for Sirt7 and GAPDH. (B) Cell lysates from Ang-II-treated CFs were immunoprecipitated with Sirt7 followed by western blotting using anti-p-Ser/Thr antibody. (C) CFs were treated with or without 100nM Ang-II for 24h followed by immunostaining with anti-Sirt7 antibody (red). (D) CFs were treated with or without 100nM Ang-II. The nuclear localization of Sirt7 was analyzed by nuclear proteins western blot. (E)CFs were transfected with empty vector or Sirt7 plasmid, and treated with or without 100nM Ang-II. The expression of α-SMA was analyzed by western blot. The expressions of α-SMA, (F) Paxillin and (G) Vinculin were analyzed by western blot. (H) CFs were transfected with negative control siRNA or Sirt7 siRNAs pool, and treated with or without 100nM Ang-II for 24h. All experiments were repeated at least three times. (*P<0.05)

In order to assess whether Sirt7 plays an important role in CFs, we first examined the effects of Ang-II on protein level of Sirt7 in CFs. Treatment with 100 nM Ang-II significantly increased Sirt7 protein levels compared to the untreated group (P<0.05) (Fig 1A and 1B). To investigate the potential role of Sirt7, we knocked down Sirt7 in CF using siRNA pools and evaluated the effect using western blotting (Fig 2A). In addition, we further found that Ang-II stimulus increased phosphorylation level of Sirt7, thereby increasing its activity (Fig 2B). In order to more intuitively show the change of Sirt7 expression, we detected the expression of Sirt7 upon Ang-II stimulation by immunofluorescence (Fig 2C). Because SIRT7 is widely expressed in cells, there is no significant intracellular localization change. To evaluate subcellular localization of Sirt7, we performed western blot analysis by using membrane, nuclear and cytoplasm fraction. The result showed that Ang-II stimulation could actually increase the nuclear localization of Sirt7 (Fig 2D). Previous study showed that Sirt7 regulates TGF-beta type 1 receptor protein levels upon TGF-β1 [17]. We examined changes in TGF-βRI/II protein levels under Ang-II stimulation (S1 Fig). The results showed that TGF-βRI/II did not show significant changes in stimulation time with 100 nM Ang-II. These indicated that different stimuli may trigger activation of different signaling pathways. All results indicated that Ang-II not only increased the protein expression of Sirt7 in a time and concentration-dependent manner different from the way of TGF-β1, but also increased its activity in CFs.

In order to examine the effect of Sirt7 on CFs to MFs differentiation, we transfected with Sirt7 and treated with or without Ang-II in CFs. Overexpression of Sirt7 could significantly increase protein levels of α-SMA and partially abrogate the effect of Ang-II-mediated upregulation of α-SMA (Fig 2E). We further examined the impact of knockdown of Sirt7 on paxillin and vinculin, which are two important adhesion-related proteins, and their changes in expression level are closely related to the morphological changes of MFs [16]. Knockdown of Sirt7 in CFs which treated with or without Ang-II displayed lower levels of expression of paxillin and vinculin (Fig 2F and 2G). In particular for vinculin, our results also demonstrate a significant trend of reduced protein expression in Sirt7 siRNA plus Ang-II-treated cells compared with Ang-II alone, even at the same level as the negative control group. When we transfected with Sirt7 siRNA pool and treated with or without Ang-II in CFs, Sirt7 depletion could partially reverse the increase in α-SMA protein expression in cells treated with or without Ang-II (Fig 2H). These results suggested Sirt7 depletion could largely reverse Ang-II induced CFs phenotypic conversion.

### 3.2. Sirt7 affects the function of cardiac fibroblasts

**Fig 3:**
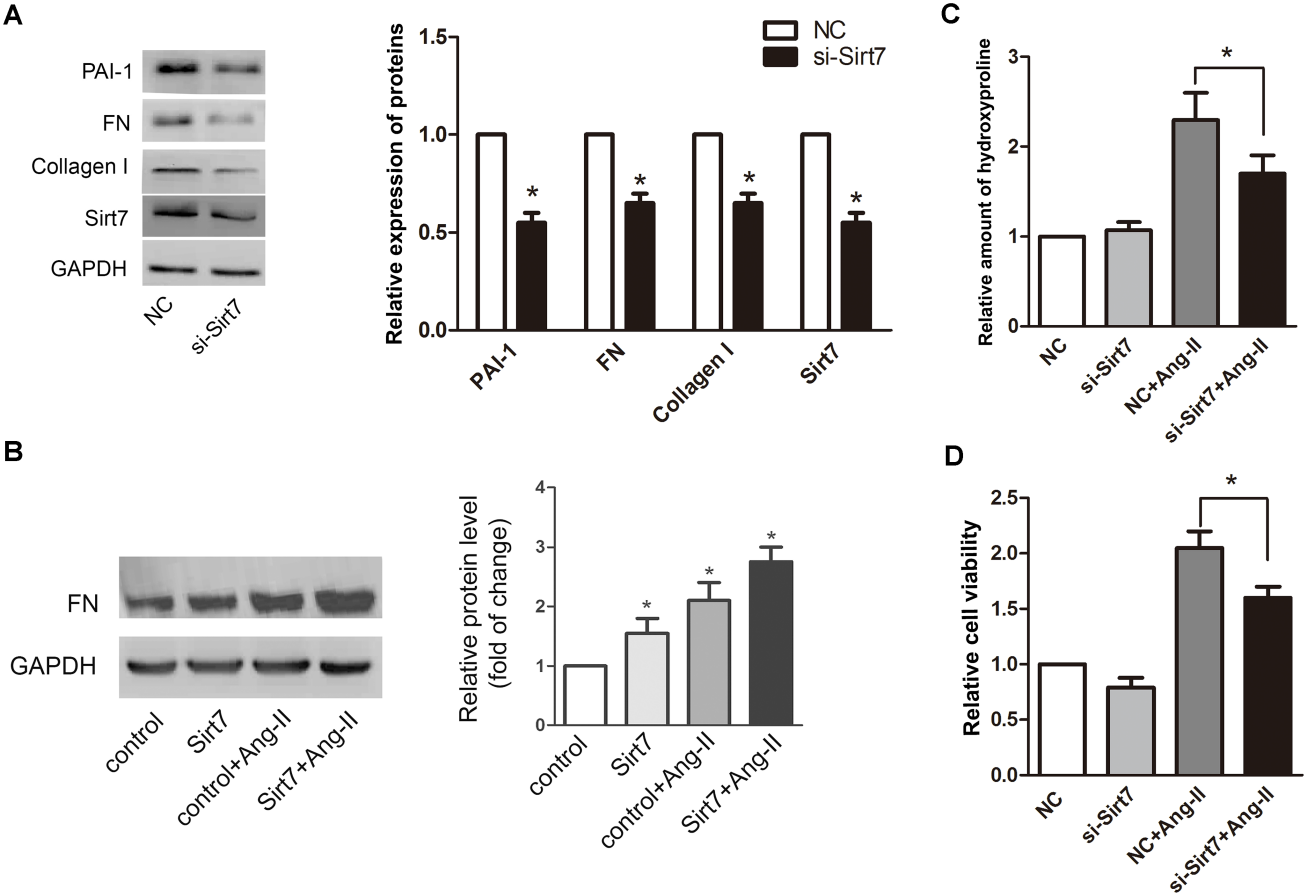
Sirt7 affects the function of CFs. (A) CFs were transfected with NC or si-Sirt7, and the expression of PAI-1, FN, Collagen I and Sirt7 were analyzed by western blot. (B) CFs were transfected with empty vector or Sirt7 plasmid, and treated with or without 100nM Ang-II. The expression of FN was analyzed by western blot. (C) CFs were transfected with NC or si-Sirt7, and treated with or without 100nM Ang-II for 24h. The hydroxyproline content was determined and analyzed. (D) CFs were transfected with NC or si-Sirt7, and treated with or without 100nM Ang-II. The cell viability was determined and analyzed. All experiments were repeated at least three times. (*P<0.05)

To investigate the potential role of Sirt7 in cardiac fibrosis, we detected some fibrotic markers in CFs, such as fibronectin (FN), collagen type I and plasminogen activator inhibitor1 (PAI-1). The results showed that knockdown of Sirt7 displayed lower protein levels of FN, collagen type I and PAI-1 (Fig 3A), and overexpression of Sirt7 could markedly increase expression of FN (Fig 3B). In addition, consistent with this notion, results show that there is a significant decrease in hydroxyproline content upon Ang-II stimulation in Sirt7-depleted CFs (Fig 3C). Knockdown of Sirt7 showed a reduced rate of proliferation upon Ang-II stimulation compared to CFs transfected with negative control siRNA (Fig 3D). All these results suggested Sirt7 depletion could reverse the increase in Ang-II-induced hydroxyproline and CF proliferation in a large extent.

### 3.3. Smad2 partially *mediates the* effects of Sirt7 on fibrosis of CFs

**Fig 4:**
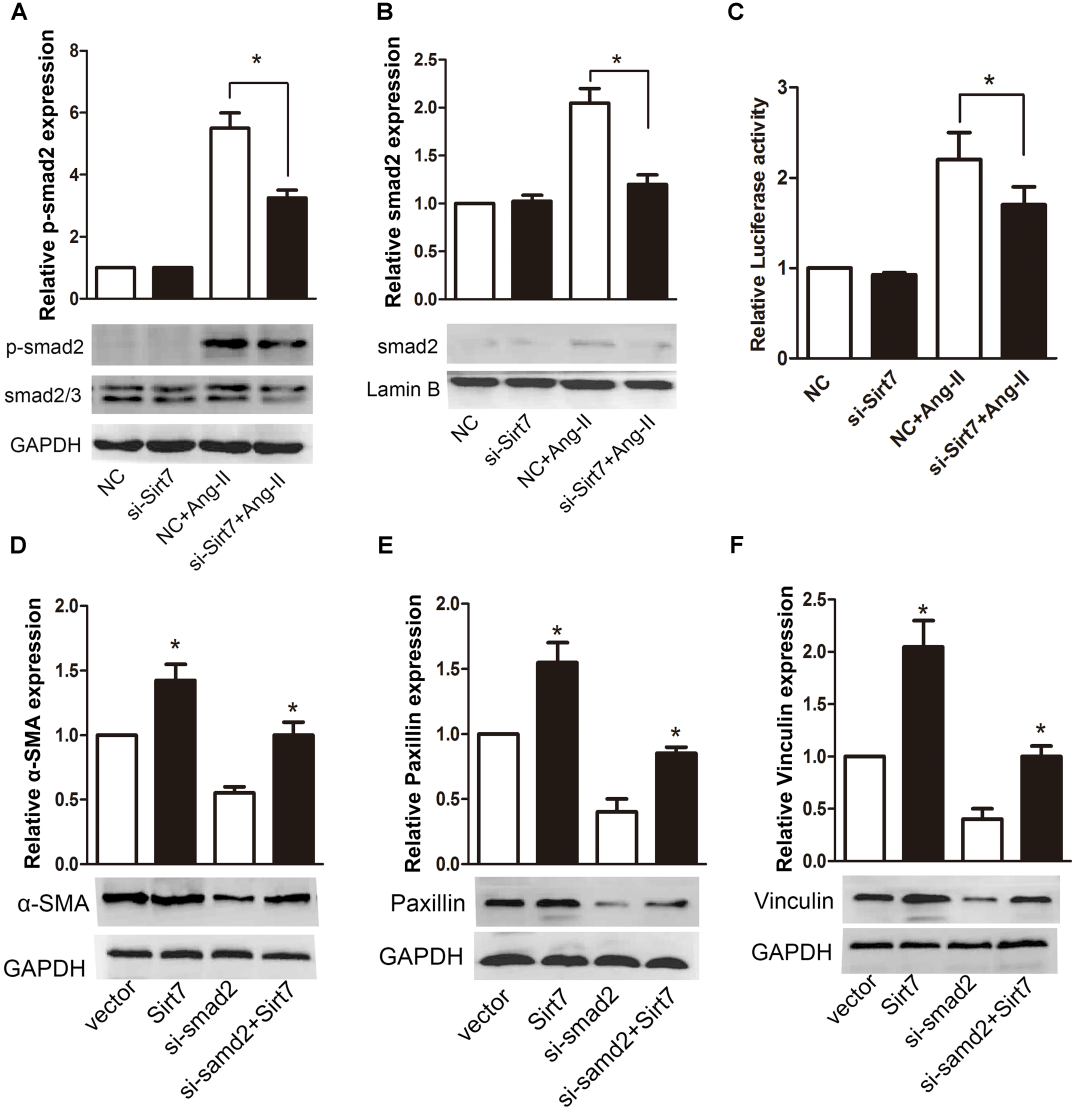
Smad2 partially mediates the effects of Sirt7 on fibrosis of CFs. (A) CFs were transfected with NC or si-Sirt7, and treated with or without 100nM Ang-II. The p-smad2 was analyzed by western blot. (B) CFs were transfected with NC or si-Sirt7, and treated with or without 100nM Ang-II. The nuclear localization of smad2 was analyzed by nuclear proteins western blot. (C) CFs were transfected with NC or si-Sirt7, and treated with or without 100nM Ang-II. The transcriptional activity of smad2 was measured through luciferase assay. (D) CFs were transfected with NC or si-samd2 and empty vector or Sirt7 plasmid. The expressions of α-SMA, (E) Paxillin and (F) Vinculin were analyzed by western blot. All experiments were repeated at least three times. (*P<0.05)

We believe that smad2 signaling may be involved in Sirt7-mediated cardiac fibrosis. In order to address this hypothesis, we examined the effect of Sirt7 depletion on smad2 phosphorylation. The result demonstrated that silencing of Sirt7 induced a significant decrease in phosphorylation level of smad2 (Fig 4A). We further examined the effect of Sirt7 on nuclear localization of smad2, because this correct positioning will affect its activity. The result showed Sirt7 depletion result in decreased nuclear localization of smad2 upon Ang-II stimulation (Fig 4B). Furthermore, we examined the effect of Sirt7 on smad2-dependent transcriptional activity by using dual-luciferase reporter gene assay. As shown, Sirt7 depletion led to decreased smad2-luciferase reporter gene activity, reversing the effect of Ang-II stimulation (Fig 4C).

We transfected CFs with smad2 siRNA pools or/and Sirt7 plasmid. As shown, overexpression of Sirt7 increased the protein expressions of α-SMA, which can be partially abrogated by smad2 siRNA (Fig 4D). Similarly, the effects of Sirt7 overexpression on paxillin (Fig 4E) and vinculin (Fig 4F) were also partially abolished by smad2 siRNA. In addition, another smad isoform, smad3, are also involved in fibroblast differentiation. So we investigated smad3 activation in CFs. The result of western blot indicated that Ang-II stimulation could indeed activate Smad3 (phosphorylation), but Ang-II stimulation could still activate smad3 after Sirt7 knockdown (S2 Fig). This indicates that the increase of smad3 activity is not mediated by Sirt7. In view of this, we did not further study the changes of smad3 in intracellular localization upon Ang-II stimulation. Taken together, these results indicate that the effect of Sirt7 on MFs transformation and even fibrosis required the participation and mediation of smad2.

### 3.4. Sirt7 regulates CFs fibrosis through activation of smad2 and ERK pathways

**Fig 5:**
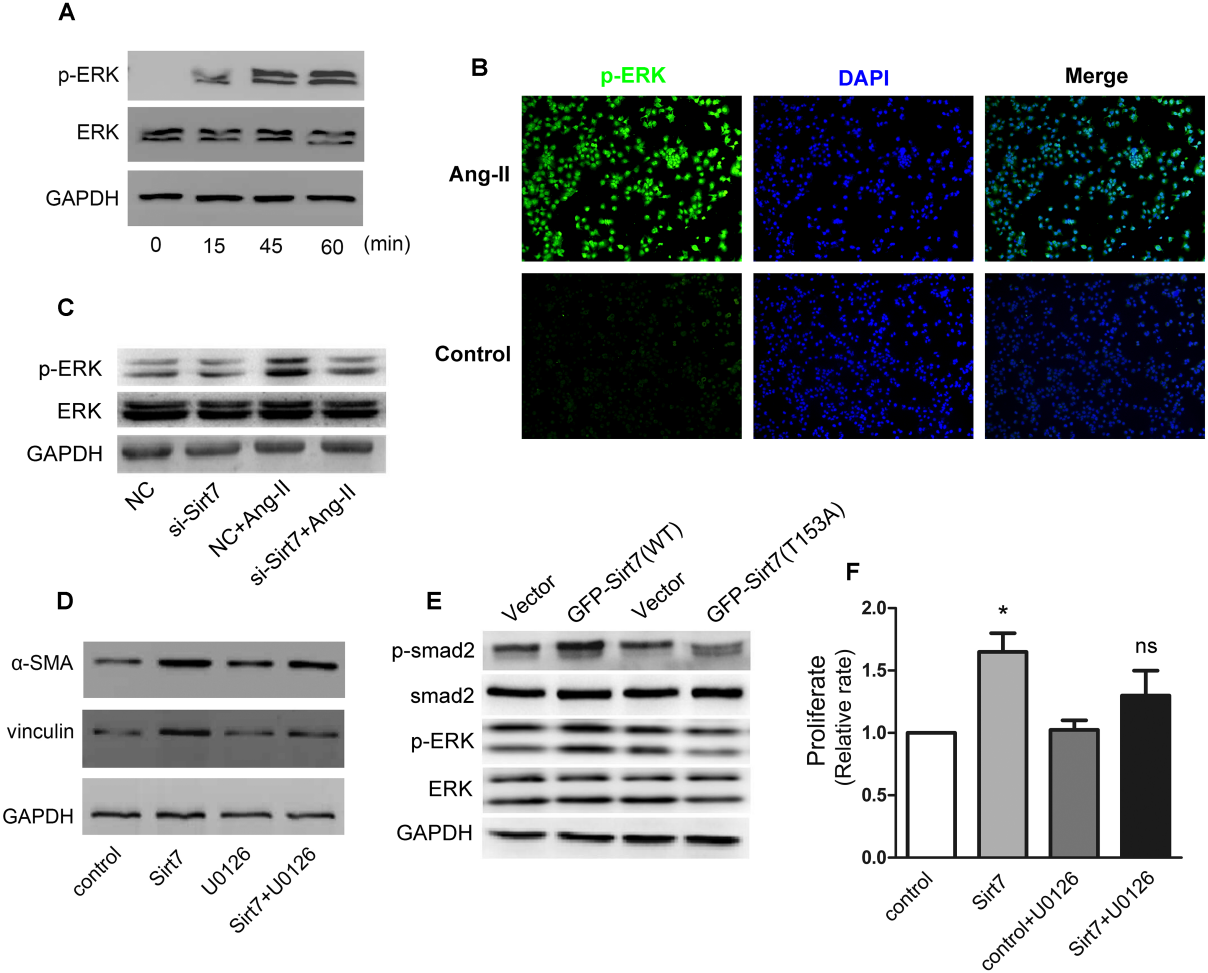
Sirt7 regulates CFs fibrosis through synergistic activation of smad2 and ERK pathways. (A) CFs were treated with Ang-II for the indicated time. The expression of p-ERK and ERK were analyzed by western blot. (B) CFs were treated with or without Ang-II followed by immunostaining with anti-p-ERK antibody (green). (C) CFs were transfected with NC or si-Sirt7, and treated with or without 100nM Ang-II. The p-ERK was analyzed by western blot. (D) CFs were treated with or without U0126 and transfected with empty vector or Sirt7 plasmid. The expressions of α-SMA and Vinculin were analyzed by western blot. (E) CFs were transfected with empty vector or Sirt7 wild-type plasmid (WT) or Sirt7 phosphorylation site mutant plasmid (Sirt7-T153A), respectively. The expression of p-ERK, ERK, p-smad2, smad2 and GAPDH were analyzed by western blot. (F) The cell proliferation was measured by CCK-8 assay. CFs were transfected with empty vector or Sirt7 plasmid, and treated with or without 100nM Ang-II. All experiments were repeated at least three times. (*P<0.05)

In view of the above effect of smad2 signaling on Sirt7-induced cardiac fibrosis-related cytology events, because the ERK signal molecules also can be used as transcription factors regulating transcription and expression of intracellular gene, we further study the effect of ERK signaling pathway on Sirt7 function. Similar to the results of smad2, Ang-II treatment leads to increase of p-ERK, but leaving total ERK constant (Fig 5A and 5B). And then, the result demonstrated that silencing of Sirt7 induced a significant decrease in p-ERK (Fig 5C). To further examined the potential role of ERK in Sirt7-depleted CFs during MFs transformation and fibrosis, we treated CFs with an inhibitor of ERK phosphorylation, U0126. The result demonstrated that overexpression of Sirt7 increased the protein expressions of α-SMA and vinculin, which can be abrogated by U0126 (Fig 5D). According to the previous study on the phosphorylation of Sirt7 (21), we constructed a Sirt7 phosphorylation site mutant plasmid (Sirt7-T153A), which was overexpressed with the Sirt7 wild-type (WT) plasmids, respectively. We examined p-smad2 and p-ERK (Fig 5E), and the results showed that overexpression of the mutant plasmid did not increase the p-smad2 and p-ERK and the overexpression of wild-type plasmids increase the p-smad2 and p-ERK. This proves that the phosphorylation of Sirt7 has a modest effect on smad2 and ERK signaling. Meanwhile, the effects of Sirt7 overexpression on proliferation of CFs were also abolished by U0126 (Fig 5F). In this, we demonstrated that Sirt7 phosphorylation is involved in the regulation of downstream signaling and cytological events through ERK and smad signaling pathway. However, it is not clear whether these two signaling pathways can promote the expression and phosphorylation of Sirt7 in turn. For this, we used the ERK inhibitor and siRNA of Smad to perform a blocking experiment to demonstrate the effect of these signaling pathways on Ang-II-induced increase of Sirt7 expression. The results showed that the two signaling pathways had no effect on the increase of Sirt7 expression (S3 Fig), suggesting that the increase in the expression of Ang-II-induced Sirt7 is mediated by other signaling pathways, whereas the increased Sirt7 is further activated through both ERK and Smad signaling pathways to promote the expression of genes related to cardiac fibrosis, as well as the molecular cytological events of CFs transformation to MFs.

## 4. Discussion

Cardiac fibrosis is a major common response of the human heart to certain pathological stimuli. Sufficiently long enough and the quantity of fibrosis is essential for the functioning of the heart and the survival of the human body. More and more research evidence indicates that sirtuin can participate in the occurrence and development of cardiovascular diseases through different aspects of function. Studies have shown that Sirt1 can protect the heart from external stress, inflammation and cardiac hypertrophy [23]; Sirt3 and Sirt7 in the heart of the stress response process also plays an extremely important role [24]. Previous studies have shown that Sirt6 can act as a negative regulator of cardiac hypertrophy, which is also indicative of its potential cardioprotective effect [25]. Based on these related studies, we speculate that SIRT7 is likely to be involved in the process of cardiac fibrosis. However, little is known about the role of Sirt7 in cardiac fibrosis. There are also studies that indicate that Sirt7 can be phosphorylated and that it is associated with many signaling pathways in certain tumor cell models [21]. However, little is known about the specific regulation of Sirt7 phosphorylation, nor is it known about the role of Sirt7 phosphorylation in cardiac fibrosis.

**Fig 6:**
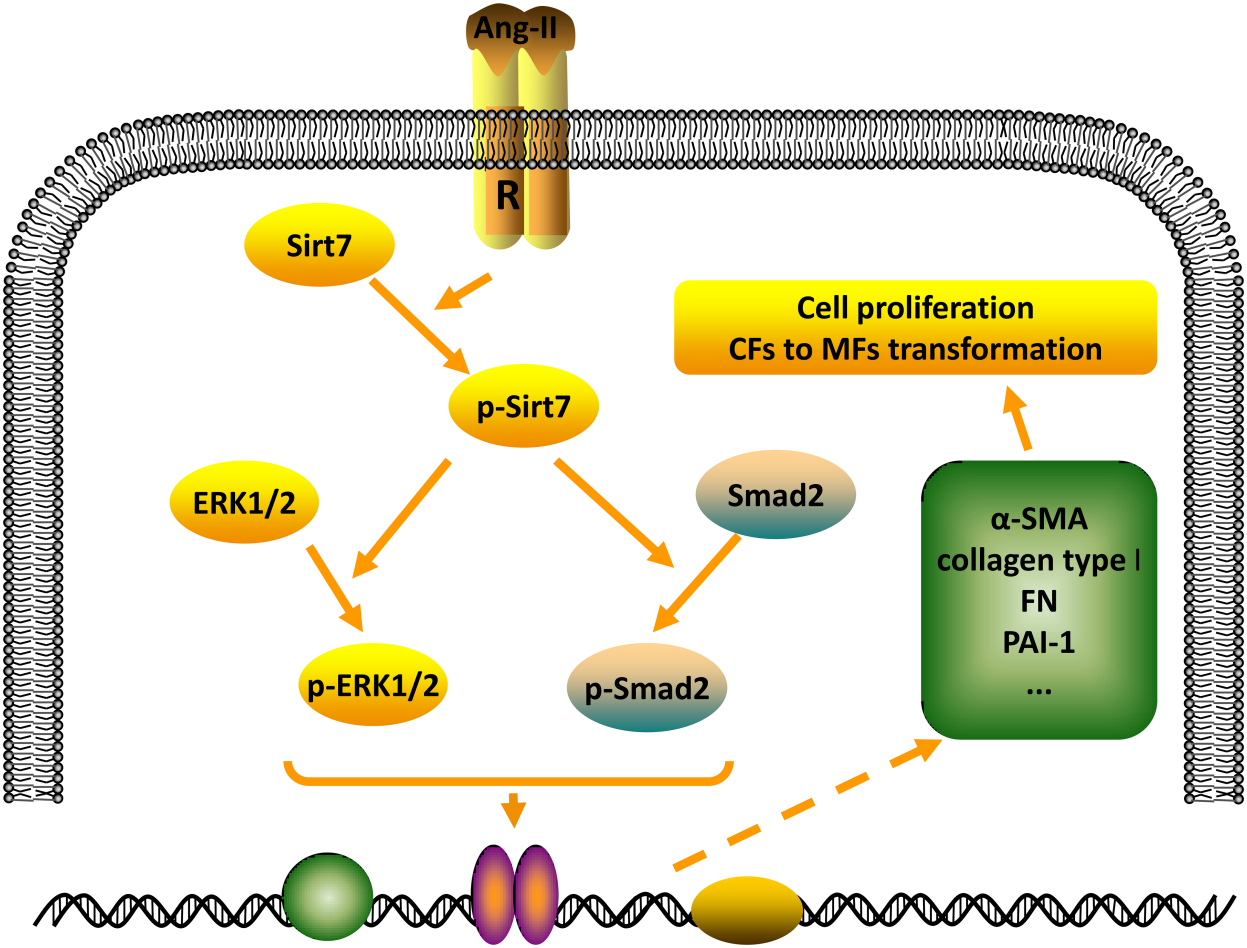
The schematic diagram illustrates the mechanism for the effect of the Ang-II/Sirt7/ERK and Ang-II/Sirt7/Smad2 axises on differentiation of CFs to MFs during cardiac fibrosis. Activation of Sirt7 by Ang-II stimulation promotes phosphorylation of ERK and Smad2. Then ERK and Smad2 regulate the activity of some transcription factors in the nucleus and further mediate expression of some genes about differentiation of CFs to MFs.

In the present study, a major finding of this study is that Sirt7 play a role in promoting fibrosis through the phosphorylation of synergistic regulation of the two signaling pathways in the process of cardiac fibrosis. During cardiac fibrosis, MFs is a highly active cell characterized by increased expression of α-SMA, production of ECM components, and formation of focal adhesions. Our results suggest that Sirt7 knockdown decreased α-SMA, ECM deposition, and up-regulated the expression of focal adhesion-associated genes and fibrosis-related genes in response to these features. And, the function of Sirt7 depends on its phosphorylation level. These also provided the first evidence that phosphorylation of Sirt7 have an impact on its activation and localization, and activated Sirt7 promotes CFs differentiation into MFs (summarized in Fig 6).

Previous studies have shown that the production of ECM components including collagen and fibronectin by CFs is induced by profibrotic factors such as Ang-II/ TGF-β which stimulates CFs phenotypic conversion to MFs [26]. In the present study, To further determine the specific molecular pathways regulating Sirt7 regulating cardiac fibrosis, we selected the ERK signaling pathway and TGF-β/smad pathway that play an important role in the organism. We have not only revealed that the expression of Sirt7 is upregulated in Ang-II stimulated CFs, but also its phosphorylation level will also be regulated by Ang-II stimulation. And, we have proved that smad2 signaling and ERK signaling commonly mediate the effects of Sirt7 on the process of cardiac fibrosis upon Ang-II stimulation.

Although the cellular and molecular processes underlying cardiac fibrosis have been largely illustrated, the strategies and targets for the treatment of cardiac fibrosis are still unsatisfactory. Here, we have reflected upon the regulatory, signaling pathway and molecular mechanisms of Sirt7 in cardiac fibrosis. Revealing a new function of Sirt7 phosphorylation activation, as well as its key role in the regulation of smad2 signaling and ERK signaling during cardiac fibrosis. An improved understanding of Sirt7 might lead to the development of new therapeutic and prognostic tools for cardiac fibrosis, thus affecting more patients worldwide.

## Supporting information

S1 Fig Effects of Ang-II stimulation on TGF-βRI/II expression. CFs were treated with 100nM Ang-II for the indicated time. The expression of TGF-βRI/II and GAPDH were analyzed by western blot.
(TIF)

S2 Fig Effects of Sirt7 on smad3 activity upon Ang-II stimulation. CFs were transfected with NC or si-Sirt7 and treated with or without 100nM Ang-II. The expression of Sirt7, p-smad3, smad3 and GAPDH were analyzed by western blot.
(TIF)

S3 Fig Effects of ERK and smad signaling pathways on Sirt7 expression. (A) CFs were treated with or without 100nM Ang-II and treated with DMSO or U0126. The expression of Sirt7, p-ERK, ERK and GAPDH were analyzed by western blot. (B) CFs were transfected with NC or si-smad2 and treated with or without 100nM Ang-II. The expression of Sirt7, smad2 and GAPDH were analyzed by western blot.
(TIF)
