# Supplementary figures and images for: Enhanced expression and phosphorylation of Sirt7 activates smad2 and ERK signaling and promotes the cardiac fibrosis differentiation upon angiotensin-II stimulation
Source: PLoS One. 2017 Jun 5;12(6):e0178530. doi: 10.1371/journal.pone.0178530 (PMC5459426; doi:10.1371/journal.pone.0178530)

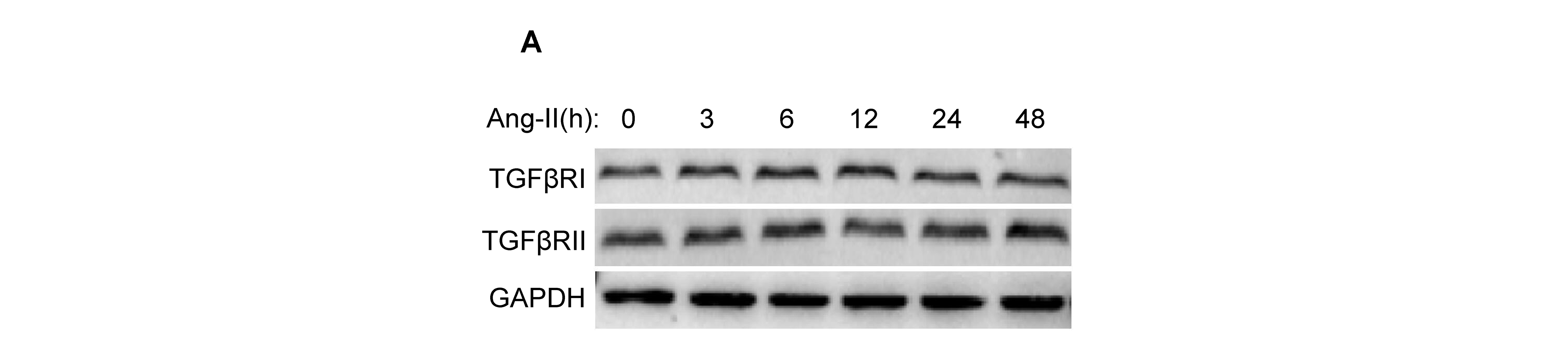

Supplement: S1 Fig — CFs were treated with 100nM Ang-II for the indicated time. The expression of TGF-βRI/II and GAPDH were analyzed by western blot. (TIF) [file pone.0178530.s001.tif]

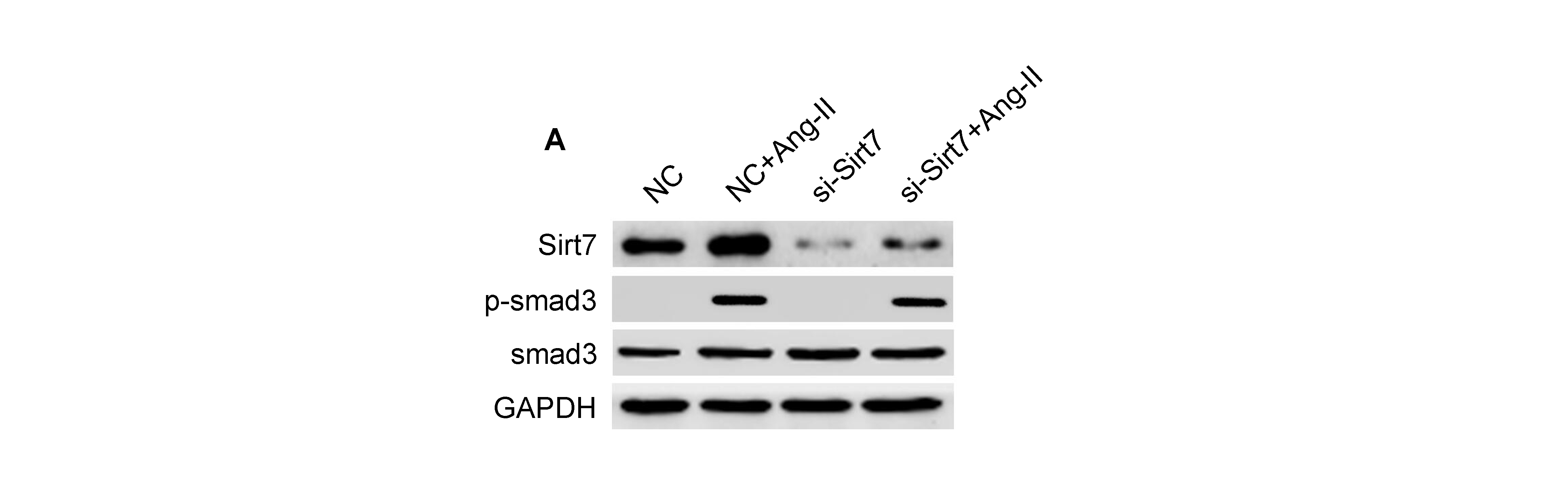

Supplement: S2 Fig — CFs were transfected with NC or si-Sirt7 and treated with or without 100nM Ang-II. The expression of Sirt7, p-smad3, smad3 and GAPDH were analyzed by western blot. (TIF) [file pone.0178530.s002.tif]

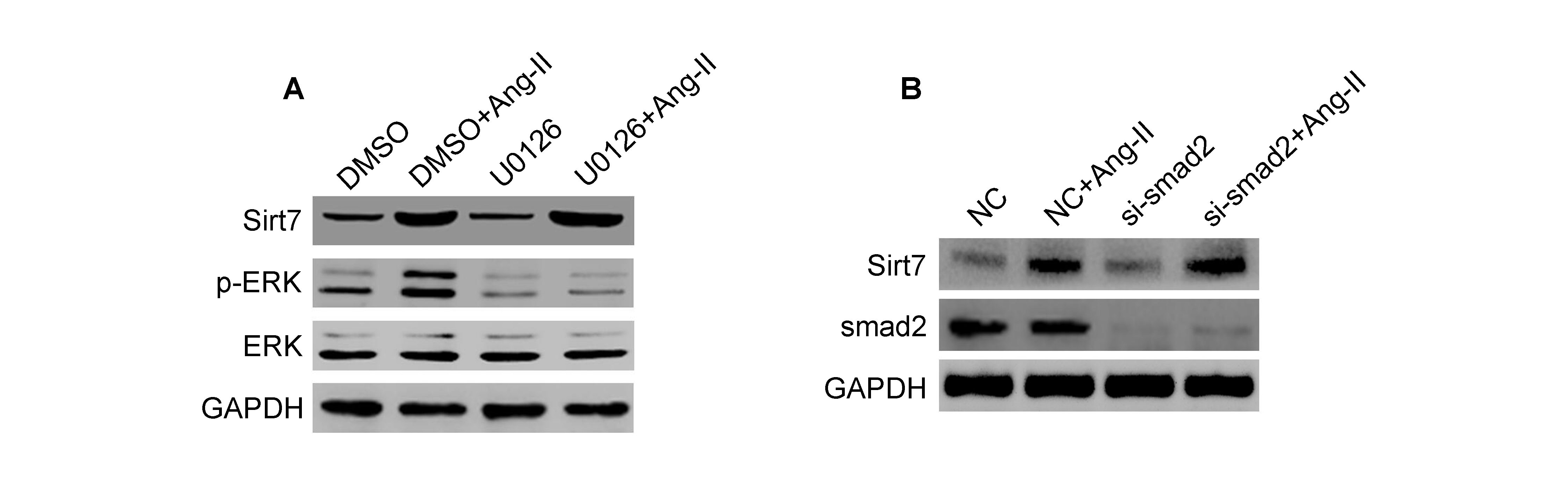

Supplement: S3 Fig — (A) CFs were treated with or without 100nM Ang-II and treated with DMSO or U0126. The expression of Sirt7, p-ERK, ERK and GAPDH were analyzed by western blot. (B) CFs were transfected with NC or si-smad2 and treated with or without 100nM Ang-II. The expression of Sirt7, smad2 and GAPDH were analyzed by western blot. (TIF) [file pone.0178530.s003.tif]
